# Transcriptome analysis of blood and spleen in virulent and avirulent mouse malaria infection

**DOI:** 10.1038/s41597-020-00592-1

**Published:** 2020-08-04

**Authors:** Yuancun Zhao, Caroline Hosking, Deirdre Cunningham, Jean Langhorne, Jing-wen Lin

**Affiliations:** 1 Division of Pediatric Infectious Diseases and Key Laboratory of Birth Defects and Related Diseases of Women and Children of MOE, State Key Laboratory of Biotherapy, West China Second University Hospital, Sichuan University, and Collaborative Innovation Center for Biotherapy, Chengdu, China; 2 Malaria Immunology laboratory, The Francis Crick Institute, London, NW1 1AT United Kingdom

**Keywords:** Parasite host response, Malaria, Transcriptomics

## Abstract

Malaria is a devastating infectious disease and the immune response is complex and dynamic during a course of a malarial infection. Rodent malaria models allow detailed time-series studies of the host response in multiple organs. Here, we describe two comprehensive datasets containing host transcriptomic data from both the blood and spleen throughout an acute blood stage infection of virulent or avirulent *Plasmodium chabaudi* infection in C57BL/6 mice. The mRNA expression profiles were generated using Illumina BeadChip microarray. These datasets provide a groundwork for comprehensive and comparative studies on host gene expression in early, acute and recovering phases of a blood stage infection in both the blood and spleen, to explore the interaction between the two, and importantly to investigate whether these responses differ in virulent and avirulent infections.

## Abstract (summary)

**Table:** 

Measurement(s)	transcriptome • gene expression • malaria
Technology Type(s)	Microarray
Factor Type(s)	blood versus spleen • virulent versus avirulent malaria infection
Sample Characteristic - Organism	Mus musculus

Machine-accessible metadata file describing the reported data: 10.6084/m9.figshare.12619232

## Background & Summary

Malaria is a mosquito-borne disease caused by *Plasmodium* parasites, inflicting nearly half a million deaths annually, mostly in low and middle income countries (World Malaria Report 2018). The deaths are mainly caused by malaria complications that particularly affect young children and pregnant women. Clinical manifestations of malaria take place during the blood stages of the infection, during which host-parasite interactions occur mainly within the vasculature and most importantly in the spleen^1,2^. As the infection progresses, the parasite also interacts with, and damages multiple host organs via the process of sequestration^3^. This adherence of infected red blood cells to the endothelium of capillaries and venues, causes complications such as cerebral malaria and acute lung injury^1,4^. Leukocytes that are tissue resident, or that are recruited into the inflamed/damaged organs are in contact with the parasite or parasite product such as hemozoin (byproduct of hemoglobin degradation)^5^ and other pathogen-associated molecular patterns (PAMPs)^6^, resulting in activation of downstream immune genes. It has long been established that spleen is the most important immune organ that generate anti-malarial immune responses^1,2^. It has no afferent lymph vessels and collects its leukocytes directly from blood. The circulating immune cells continuously migrate into and out of the spleen, with their changed transcriptional activities during the course of malaria infection. In support of this, a recent study showed that parasite specific CD8^+^ T cell were primed in the spleen and migrated into the lungs^7^; another study showed that the ‘lung pathology’ signature can be picked up by analysing whole blood transcriptome^8^.

Genome-wide expression profiling is being increasingly applied to dissect the complex details of the host response to malaria infection^9,10^. As blood is the most accessible tissue in field studies, numerous field studies analyse blood transcriptomes as read-outs for anti-malarial immunity^11–13^. Therefore, it is very important to understand whether the immune responses detected in the blood serve as a reliable proxy for immune responses occurring in the spleen; if so, to what extent and at which stage of infection are they most closely related. To date, few studies have performed transcriptomic analyses of the blood in the mouse model^9^ and only one study carried out by us attempted to investigate the similarities between blood and spleen transcriptome^14^.

**Fig. 1:**
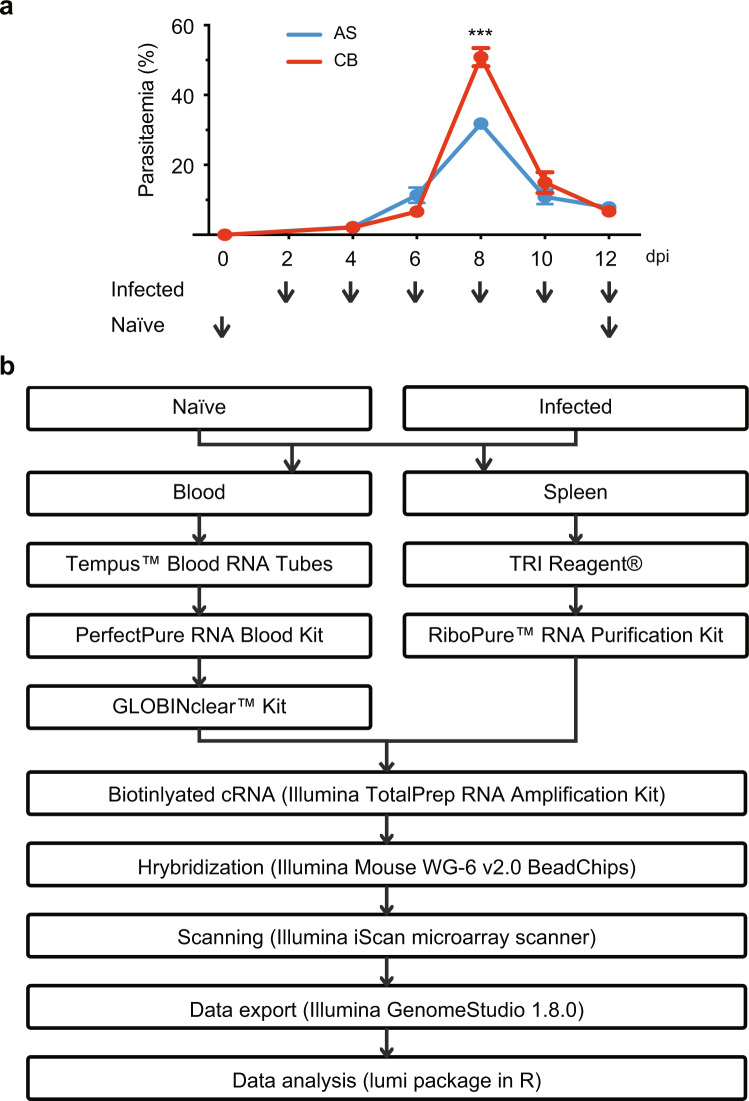
Sample collection and workflow. **(a)** Parasitemia (percentage of infected erythrocytes) of infected mice during the acute phase of blood stage infection and the time points (arrow heads) when the samples were collected. The mice were intraperitoneally infected with 10^5^ erythrocytes that were infected with *P. chabaudi* parasite. **(b)** The flow chart illustrating the steps of microarray analysis.

Here we describe two comprehensive, time-series analyses of the blood and spleen transcriptomic changes throughout the acute phase of blood stage infection (Fig. 1a) using a well-established rodent malaria model, *Plasmodium chabaudi*. This parasite is widely used to study host responses as it mirrors many pathological manifestations associated with *P. falciparum* infection, the most deadly species infecting humans, including parasite sequestration, severe malarial anemia, and chronic infection^8,15,16^. Time-series gene expression analysis is most helpful in identifying genes with transient expression changes and in investigation of gene regulation profiles during an infection. In our previous studies, we showed that pathology signatures can be picked up from blood transcriptome and they are quite distinct in the avirulent *P. chabaudi* AS or virulent *P. chabaudi* CB infection^8^; further analysing the blood and spleen transcriptome from the avirulent *P. chabaudi* AS infection, we identified only a small set of immune genes shared between them^14^. Here we report a new dataset of spleen transcriptome from the virulent *P. chabaudi* CB infection, which were collected from the same mice as the published blood transcriptome^8^. Our datasets, including the published PcAS/PcCB blood^8^, PcAS spleen^14^ and this new PcCB spleen transcriptome, offer a unique possibility to identify the complete set of activated or suppressed genes during an acute blood stage infection, to infer their rates of change and their causal effects. Further, it would be of high interest to investigate whether the interaction between blood and spleen differ in these two infections or whether more subtle relationship can be unearthed using more elaborate time modeling methods.

## Methods

### Mice and parasites

Female C57BL/6 aged 6–8 weeks from the SPF (Specific Pathogen Free) unit at the Francis Crick Institute Mill Hill Laboratory were housed under reverse light conditions (light 19.00–07.00, dark 07.00–19.00 GMT) at 20–22 °C, and were allowed access to diet and water *ad libitum*. This study was carried out in accordance with the UK Animals (Scientific Procedures) Act 1986 (Home Office license 80/2538 and 70/8326), and was approved by the Francis Crick Institute Ethical Committee.

Cloned lines of *Plasmodium chabaudi chabaudi* AS and CB were originally obtained from David Walliker, University of Edinburgh, United Kingdom. Infections were initiated by intraperitoneal injection of 10^5^ parasitised erythrocytes derived from cryopreserved stocks. The course of infection was monitored on Giemsa-stained thin blood films by enumerating the percentage of erythrocytes infected with asexual parasites (parasitemia). The limit of detection for patent parasitemia was 0.01% infected erythrocytes. During the experiments, mouse condition were closely monitored. Core body temperature was measured with an infrared surface thermometer (Fluke); body weight was calculated relative to a baseline measurement taken before infection; and erythrocyte density was determined on a VetScan HM5 haematology system (Abaxis). The animals were euthanized upon reaching humane end points by showing the following signs: emaciation (more than 25% weight loss), persistent labored breathing, severe hypothermia (body temperature below 28 °C), inability to remain upright when conscious or lack of natural functions, or continuous convulsions lasting more than 5 min.

### RNA isolation and preparation for microarray analysis

The sample collection and processing workflow is summarised in Fig. 1. These methods are expanded versions of descriptions in our related studies^8,14^. Female C57BL/6 mice aged between 6–8 weeks were injected intraperitoneally with 10^5^ infected red blood cells of *P. chabaudi* AS or CB strain. At 2, 4, 6, 8, 10 and 12 days post infection (dpi), 0.5 mL of blood was collected via cardiac puncture into 1 mL Tempus RNA stabilising solution (Applied Biosystems). Spleens were aseptically removed and were homogenised immediately in TRI reagent (Ambion) by pulsing with a Polytron homogenising unit (Kinematic). An extra day 9 group was collected from PcCB infected mice that had reached humane end points. Naïve control samples were also collected on day 0 (the day of infection) and day 12 (the end of the experiment). Samples were snap frozen in dry ice and stored at −80 °C until RNA isolation.

Total blood RNA was extracted using PerfectPure RNA Blood Kit (5 PRIME), and Globin mRNA was removed from 2 µg of total isolated RNA using GLOBINclear 96-well Mouse/Rat Whole Blood Globin Reduction Kit (Ambion) according to the manufacturer’s instructions. Total splenic RNA was extracted using RiboPure RNA Purification Kit (Ambion) following the manufacture’s protocol. All RNA samples derived from the same experiment were isolated altogether at the end of the experiment, in 2–3 batches within a day. Globin mRNA reduction was performed in 2 batches, one for all PcAS blood samples and the other for all PcCB blood. Batch information for RNA isolation and subsequent processing was provided in Online-only Tables 1–4.

To test whether parasite RNA gives signals in microarray analysis of mouse gene expression, an independent experiment was performed using naïve and infected mouse blood RNA, and purified parasite RNA. RNA from naïve or infected blood was processed as described above. Parasite purification and RNA extraction methods were performed as described previously^17^. Briefly, infected blood collected at day 8 post infection were depleted of leukocytes by filtration through Plasmodipur filters (EuroProxima) followed by erythrocyte lysis using 0.15% saponin (Sigma) in ice-cold PBS and extensive washes with PBS. Purified parasite pellets were then resuspended in 1 ml TRI reagent (Ambion), snap-frozen on dry ice and kept at −80 °C. Parasite RNA was extracted using RiboPure RNA Purification Kit (Ambion) according to the manufacturer’s protocols. Parasite RNA was also subjected to Globin mRNA removal. Batch information for RNA isolation and processing was provided in Online-only Table 5.

Biotinylated, amplified antisense complement RNA (cRNA) samples were prepared from 300 ng of either globin reduced blood/parasite RNA, or splenic total RNA using Illumina TotalPrep RNA Amplification Kit (Ambion). cRNA was prepared in 4 batches: PcAS blood, PcCB blood, all spleen samples and blood/parasite RNA.

At each step, the quantity of the RNA samples was measured using NanoDrop 1000 Spectrophotometer (Thermo Fisher Scientific) and the quality of RNA was verified using Agilent 2100 bioanalyzer (Agilent Technologies) or Caliper LabChip GX (Caliper Life Sciences), provided as RNA Integrity Number (RIN) or RNA Quality Score (RQS), respectively. Only RNA samples with RIN/RQS above 7 were used for subsequent treatment and analysis; however, for blood samples with high parasite load, parasite rRNA affected RIN/RQS determination, and the quality of these samples were determined by examining electropherograms. RNA concentration and RIN/RQS of each sample were also provided in Online-only Tables 1–5.

### Microarray hybridisation and raw data export

The following procedures for microarray hybridisation and data acquisition was done for each sample. Briefly, 1.5 µg of labelled cRNA was hybridised to Illumina Mouse WG-6 v2.0 Expression BeadChip (consisting of 45,281 probe sets representing 30,854 genes) according to the manufacturer’s protocols. The arrays were then washed, blocked, stained and scanned on an Illumina iScan, following the manufacturer’s instructions. Illumina BeadStudio/GenomeStudio 1.8.0 software was used to generate signal intensity values, quality control values, and to subtract background. Hybridisation was performed in 4 batches: PcAS blood, PcCB blood, all spleen samples and blood/parasite RNA.

### Microarray data preparation and analysis

Data input, quality control, variance stabilisation, log transformation and quantile normalisation were performed using the lumi package^18^. The full feature set (a total of 45,281 probes) of each sample was used for the following analyses including hierarchical clustering, principle component analysis (PCA) and Euclidean distance, all conducted using R 3.6.0 (www.r-project.org). For hierarchical clustering, agglomerative clustering with average linkage was used.

## Data Records

Gene expression data were deposited at the Gene Expression Omnibus database (GEO) under the following accession numbers: GSE93631^19^ (AS and CB blood) and GSE123391^20^ (AS spleen) which were published previously^8,14^; GSE145781^21^ (CB spleen) and GSE145634^22^ (the raw data of parasite RNA experiment) which were new datasets.

GEO accession numbers of blood or spleen samples that were derived from the same mouse were provided in Tables 1 and 2. Batch information, RNA quality and concentration and related GEO accession numbers were provided in Online-only Tables 1–5.

**Table 1:** GEO accession numbers of blood or spleen samples that were derived the same PcAS infected mouse (Data Source: GSE93631^19^ and GSE123391^20^).

mouse No.	Blood (GSE93631^19^)		Spleen (GSE123391^20^)	
	GEO accession	BeadChip No.	GEO accession	BeadChip No.
naïve_D0_m1	GSM2459164	8762536135_E	GSM3502544	9440690022_B
naïve_D0_m2	GSM2459165	8762536084_A	GSM3502545	9440690030_C
naïve_D0_m3	GSM2459166	8762536052_F	GSM3502546	9440690035_B
naïve_D12_m1	GSM2459167	8762536072_E	GSM3502568	9440690030_F
naïve_D12_m2	GSM2459168	8784170061_E	GSM3502569	9440690037_C
naïve_D12_m3	GSM2459169	8784170059_F	GSM3502570	9440690042_A
AS_D2_m1	GSM2459172	8762536135_C	GSM3502547	9440690022_C
AS_D2_m2	GSM2459171	8762536084_B	GSM3502548	9440690035_A
AS_D2_m3	GSM2459173	8784170059_C	GSM3502549	9440690037_D
AS_D2_m4	GSM2459170	8762536072_F	GSM3502550	9440690042_C
AS_D4_m1	N/A	N/A	GSM3502551	9440690022_D
AS_D4_m2	GSM2459175	8762536084_F	GSM3502552	9440690030_A
AS_D4_m3	GSM2459174	8762536052_B	GSM3502553	9440690037_E
AS_D4_m4	GSM2459176	8784170061_A	GSM3502554	9440690042_B
AS_D6_m1	N/A	N/A	GSM3502555	9440690022_E
AS_D6_m2	GSM2459177	8762536052_E	GSM3502556	9440690035_C
AS_D6_m3	GSM2459179	8784170059_B	GSM3502557	9440690042_D
AS_D6_m4	GSM2459178	8762536135_A	GSM3502558	9440690037_A
AS_D8_m1	GSM2459182	8784170059_E	GSM3502559	9440690022_F
AS_D8_m2	GSM2459181	8762536135_F	GSM3502560	9440690030_B
AS_D8_m3	GSM2459180	8762536084_D	GSM3502561	9440690035_D
AS_D10_m1	GSM2459185	8784170061_B	GSM3502562	9440690035_E
AS_D10_m2	GSM2459184	8762536135_D	GSM3502563	9440690030_D
AS_D10_m3	GSM2459183	8762536052_C	GSM3502564	9440690037_F
AS_D12_m1	GSM2459186	8762536072_C	GSM3502565	9440690030_E
AS_D12_m2	GSM2459188	8784170061_C	GSM3502566	9440690035_F
AS_D12_m3	GSM2459187	8762536084_E	GSM3502567	9440690037_B

**Table 2:** GEO accession numbers of blood or spleen samples that were derived the same PcCB infected mouse (Data Source: GSE93631^19^ and GSE145781^21^).

mouse No.	Blood (GSE93631^19^)		Spleen (GSE145781^21^)	
	GEO accession	BeadChip No.	GEO accession	BeadChip No.
naïve_D0_m1	GSM2459189	8762536055_D	GSM4332890	9440690042_E
naïve_D0_m2	GSM2459190	8762536056_E	GSM4332891	9440690065_D
naïve_D0_m3	GSM2459191	8762536079_E	GSM4332892	9440690046_F
naïve_D12_m1	GSM2459192	8762536054_F	GSM4332893	9440690056_F
naïve_D12_m2	GSM2459193	8762536055_E	GSM4332894	9440690059_B
naïve_D12_m3	GSM2459194	8762536056_A	GSM4332895	9440690061_A
CB_D2_m1	GSM2459195	8762536049_B	GSM4332896	9440690056_B
CB_D2_m2	GSM2459196	8762536054_C	GSM4332897	9440690046_A
CB_D2_m3	GSM2459197	8762536055_F	GSM4332898	9440690061_F
CB_D2_m4	GSM2459198	8762536079_C	GSM4332899	9440690059_C
CB_D4_m1	GSM2459199	8762536049_F	GSM4332900	9440690046_B
CB_D4_m2	GSM2459200	8762536054_D	GSM4332901	9440690061_C
CB_D4_m3	GSM2459201	8762536056_B	GSM4332902	9440690056_E
CB_D4_m4	GSM2459202	8762536078_A	GSM4332903	9440690065_F
CB_D6_m1	GSM2459206	8762536097_B	GSM4332904	9440690046_C
CB_D6_m2	GSM2459203	8762536055_A	GSM4332905	9440690042_F
CB_D6_m3	GSM2459204	8762536056_F	GSM4332906	9440690056_A
CB_D6_m4	GSM2459205	8762536079_A	GSM4332907	9440690061_B
CB_D8_m1	GSM2459207	8762536049_D	GSM4332908	9440690056_C
CB_D8_m2	GSM2459208	8762536054_E	GSM4332909	9440690065_A
CB_D8_m3	GSM2459209	8762536078_D	GSM4332910	9440690061_E
CB_D8_m4	GSM2459210	8762536097_F	GSM4332911	9440690059_D
CB_D9_m1	GSM2459221	8762536054_A	GSM4332912	9440690065_C
CB_D9_m2	GSM2459218	8762536055_B	GSM4332913	9440690059_E
CB_D9_m3	GSM2459219	8762536097_D	N/A	N/A
CB_D9_m4	GSM2459220	8762536078_F	GSM4332914	9440690061_D
CB_D10_m1	GSM2459211	8762536049_C	GSM4332915	9440690065_E
CB_D10_m2	GSM2459212	8762536056_C	GSM4332916	9440690056_D
CB_D10_m3	GSM2459213	8762536078_B	GSM4332917	9440690046_E
CB_D10_m4	GSM2459214	8762536079_D	GSM4332918	9440690059_A
CB_D12_m1	GSM2459215	8762536049_E	GSM4332919	9440690059_F
CB_D12_m2	GSM2459216	8762536078_C	GSM4332920	9440690046_D
CB_D12_m3	GSM2459217	8762536079_B	GSM4332921	9440690065_B

## Technical Validation

### Sample preparations and quality control

**Fig. 2:**
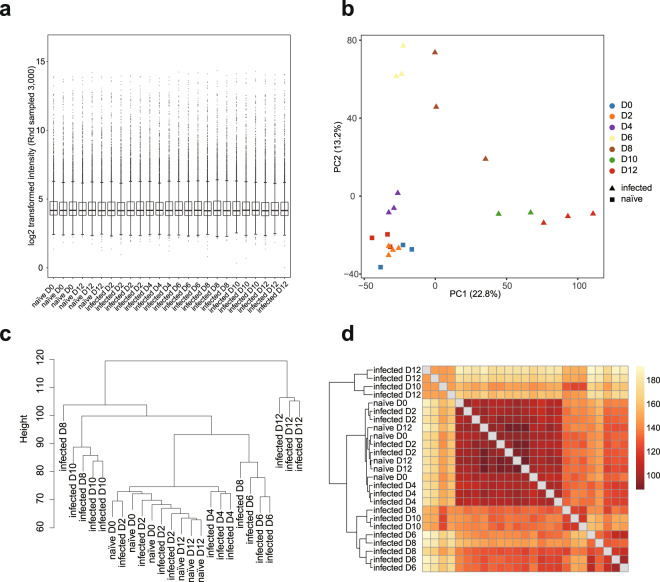
Quality check of BeadChip gene expression data of PcAS blood samples. (**a**) Box plot showing distribution of 3,000 randomly sampled probe signals for normalised PcAS infected blood expression data. The median, two hinges, two whiskers and outlying points were shown. (**b**) Principal component analysis of normalised expression data of naïve and infected blood samples. (**c**) Hierarchical clustering plot of normalised intensity data among the samples was generated using agglomerative clustering with average linkage. (**d**) Heatmap of Euclidean distance. A full feature set was used for (**b**–**d**). This dataset was submitted to GEO (GSE93631).

**Fig. 3:**
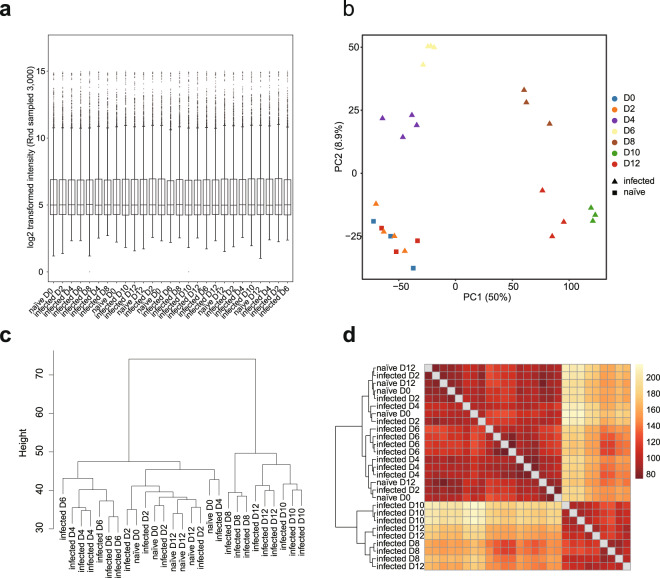
Quality check of BeadChip gene expression data of PcAS spleen samples.(**a**) Box plot showing distribution of 3,000 randomly sampled probe signals for normalised PcAS spleen expression data. The median, two hinges, two whiskers and outlying points were shown. (**b**) Principal component analysis of normalised expression data of naïve and infected spleen samples. (**c**) Hierarchical clustering plot of normalised intensity data among the samples was generated using agglomerative clustering with average linkage. (**d**) Heatmap of Euclidean distance. A full feature set was used for (**b-d**). This dataset was submitted to GEO (GSE123391).

Several aspects of the experiment were designed to ensure the quality of the data. For example, the control naïve mice were randomly selected from the same batch of age-matched mice, 3 of which were sacrificed at the same day of infection, and 3 of which were housed under the same conditions as the infected mice and were sacrificed along with mice after 12 days of infection. All mice in the infected group were infected at the same time and were randomly selected for sample collection at each time point. Overall, both blood and spleen samples collected from either PcAS or PcCB infections showed uniformed normalised intensities (Figs. 2a and 3a). Importantly, high similarities were observed between biological replicates (Figs. 2 and 3).

### Quality check of time dependent responses

**Fig. 4:**
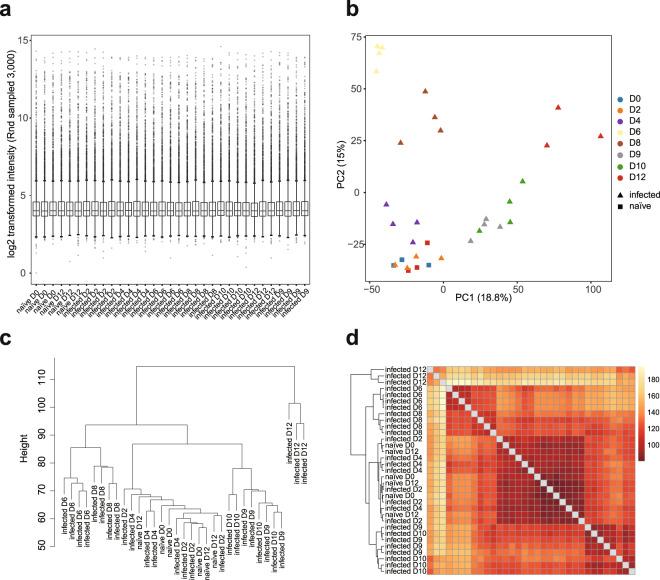
Quality check of BeadChip gene expression data of PcCB blood samples. (**a**) Box plot showing distribution of 3,000 randomly sampled probe signals for normalised PcCB blood expression data. The median, two hinges, two whiskers and outlying points were shown. (**b**) Principal component analysis of normalised expression data of naïve and infected blood samples. (**c**) Hierarchical clustering plot of normalised intensity data among the samples was generated using agglomerative clustering with average linkage. (**d**) Heatmap of Euclidean distance. A full feature set was used for (**b-d**). This dataset was submitted to GEO (GSE93631).

**Fig. 5:**
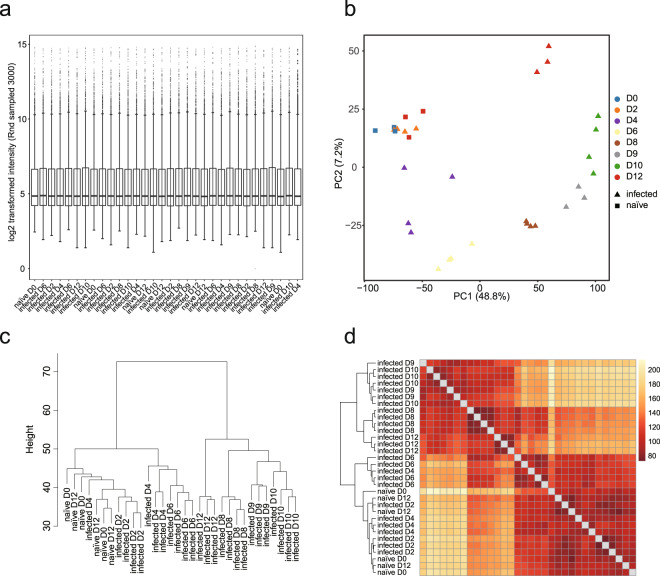
Quality check of BeadChip gene expression data of PcCB spleen samples. (**a**) Box plot showing distribution of 3,000 randomly sampled probe signals for normalised PcCB spleen expression data. The median, two hinges, two whiskers and outlying points were shown. (**b**) Principal component analysis of normalised expression data of naïve and infected spleen samples. (**c**) Hierarchical clustering plot of normalised intensity data among the samples was generated using agglomerative clustering with average linkage. (**d**) Heatmap of Euclidean distance. A full feature set was used for (**b-d**). This dataset was submitted to GEO (GSE145781).

In the naïve control group, mice collected at day 0 or day 12 clustered together in all 4 datasets. Interestingly, samples collected at 2 dpi at which time point the infection rate was below microscopic detection level, also cluster with naïve groups; and this was observed in both the blood and spleen in either infection (Figs. 2–5). In the avirulent PcAS infection, from day 4 onwards, the expression profiles changed significantly, showing clear time-dependent responses in both the blood and the spleen. At 4 dpi, spleen showing longer distance from the naïve groups than the blood, 46.4 vs 21.6 distance on PC2 (Figs. 2b and 3b), which indicates higher host responses in the spleen than in the blood. Similar responses took place in the virulent PcCB infection, showing 4 dpi-naïve distance on PC2 of 18.5 in the blood vs 32.0 in the spleen (Figs. 4b and 5b). This is in line with the current view that parasite-host interaction mainly take place in the spleen.

An interesting difference between the blood and spleen is the divergence between day 6 and 8 post infection. In the avirulent PcAS infection the distances between 6 and 8 dpi on PC1 were 36.4 in the blood and 91.8 in the spleen (Figs. 2b and 3b). This is slightly less striking in the virulent PcCB infection, with 21.1 in the blood and 66.8 in the spleen. These differences are also apparent in hierarchical clustering and heatmaps of euclidean distance (Figs. 2 and 3).

The striking differences between PcAS and PcCB infections were the responses took place between day 10 and 12 post infection. In the avirulent PcAS infection, while day 10 and 12 were clearly different from previous infected samples, they clustered tightly together in both blood and spleen samples (Figs. 2b and 3b). By contrast, in the virulent PcCB infection the two days differed in both PC1 and PC2 (Figs. 4b and 5b), and the heatmaps of Euclidean distance showed that 12 dpi clearly separate from other samples (Figs. 4d and 5d).

### Parasite RNA does not affect BeadChip gene expression results

**Fig. 6:**
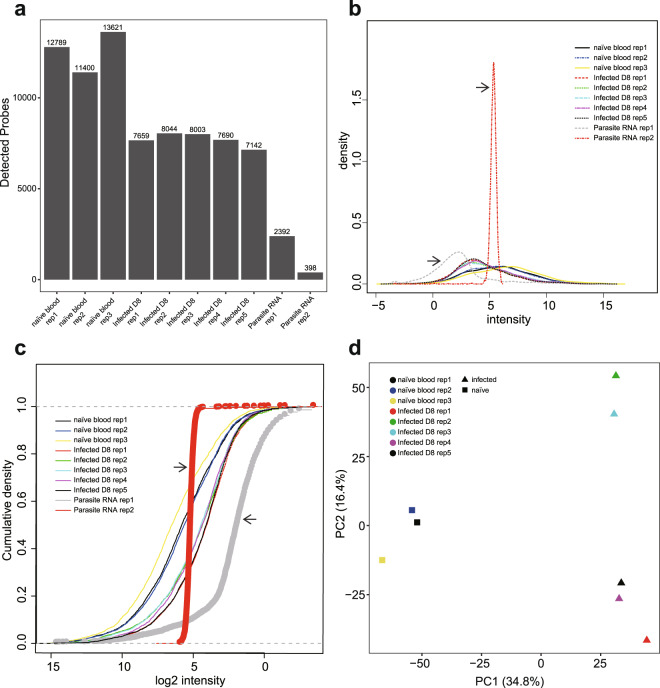
Validation of parasite RNA does not affect BeadChip gene expression results. (**a**) Bar chart showing the number of probes detected in each sample. (**b**) Density plot of non-normalised expression data showing the signal density distribution. (**c**) Cumulative distribution function plot of non-normalised expression data of each sample. Arrowheads indicate parasite samples. (**d**) PCA plot of normalised expression data from infected and naïve blood samples after excluding parasite samples. This dataset was submitted to GEO (GSE145634).

Because the malaria parasite infects erythrocytes, RNA isolated from the infected blood contains both mouse and *Plasmodium* RNA. We therefore performed an independent experiment to rule out the interference of parasite RNA in downstream analysis using Mouse WG-6 v2.0 Expression BeadChip. We prepared purified *P. chabaudi* AS parasite RNA by passing infected blood through a leukocyte filter, usually removing more than 99% leukocytes, followed by erythrocyte lysis and extensive washes. Globin mRNA removal was also performed as for infected blood samples. As shown in Fig. 6, the numbers of detectable probes in parasite samples were significantly lower (Fig. 6a), and this hindered the normalisation step. Moreover, the non-normalised expression data of parasite samples showed very different density or cumulative density profiles (Fig. 6b,c). After removing parasite data from the dataset, the subsequent analyses can be easily performed and it was clear that the infected blood collected at 8 dpi significantly differed from naïve blood (Fig. 6d), validating our previous finding.

## Usage Notes

One major advantage of this study is that we collected both the blood and spleen simultaneously from the same mouse (GEO accession numbers of blood or spleen samples that were derived from the same mouse were provided in Tables 1 and 2) throughout the acute phase of blood stage infection, from as early as day 2 post infection when the infection rate was below microscopic detection, till day 12 post infection when the parasite load was controlled. Moreover, the samples were collected at 2-day intervals to allow a more detailed analysis of the time-dependent transcriptional changes. It is hoped that this will facilitate the users to investigate in detail the interaction between the blood and the spleen. It would also provide some answers to the question of whether some of the responses in the blood happen before or after the spleen responses, for example using time series modelling. And importantly, we collected samples from both the virulent PcCB and the avirulent PcAS infections. It would be of high interest to investigate whether the interaction between blood and spleen differ in these two infections.

## Data Availability

R scripts for raw data reading, normalisation, QC, and plotting were available at https://github.com/LuChenLab/Rscript_for_BeadChip.git.

## Online-only Tables

**Online-only Table 1:** Batch information, RNA quality and concentration and related GEO accession numbers for PcAS blood (GSE93631^19^).

Series GSE93631	PRJNA361313		Caliper LabChip		Nanodrop						
GEO accession ID	Sample Name in GEO	BeadChip No.	RQS	rRNA 28 s/18 s	[ng/ul]	260/280	260/230	RNA isolation batch	Globin mRNA reduction	cRNA preparation	BeadChip hybridasation
GSM2459164	AS_naive_D0 rep 1	8762536135_E	9	2.85	147.7	2.1	2.25	03/06/2013_Batch1	In one batch	In one batch	In one batch
GSM2459165	AS_naive_D0 rep 2	8762536084_A	8.2	2.27	164.28	2.12	2.24	03/06/2013_Batch1	In one batch	In one batch	In one batch
GSM2459166	AS_naive_D0 rep 3	8762536052_F	8.3	2.33	244.74	2.1	2.24	03/06/2013_Batch1	In one batch	In one batch	In one batch
GSM2459167	AS_naive_D12 rep 1	8762536072_E	8.2	2.18	325.34	2.1	2.22	03/06/2013_Batch1	In one batch	In one batch	In one batch
GSM2459168	AS_naive_D12 rep 2	8784170061_E	8	2.03	372.4	2.08	2.23	03/06/2013_Batch1	In one batch	In one batch	In one batch
GSM2459169	AS_naive_D12 rep 3	8784170059_F	8.3	2.13	305.17	2.1	2.17	03/06/2013_Batch1	In one batch	In one batch	In one batch
GSM2459170	AS_D2 rep 1	8762536072_F	8.7	2.58	210.18	2.13	2.2	03/06/2013_Batch1	In one batch	In one batch	In one batch
GSM2459171	AS_D2 rep 2	8762536084_B	7.6	2.62	343.31	2.09	2.21	03/06/2013_Batch1	In one batch	In one batch	In one batch
GSM2459172	AS_D2 rep 3	8762536135_C	6.9	3.32	269.34	2.1	2.28	03/06/2013_Batch1	In one batch	In one batch	In one batch
GSM2459173	AS_D2 rep 4	8784170059_C	7.5	2.8	504.89	2.17	2.27	03/06/2013_Batch1	In one batch	In one batch	In one batch
GSM2459174	AS_D4 rep 1	8762536052_B	7.9	1.9	314.44	2.12	2.26	03/06/2013_Batch1	In one batch	In one batch	In one batch
GSM2459175	AS_D4 rep 2	8762536084_F	7.9	1.91	315.67	2.1	2.26	03/06/2013_Batch1	In one batch	In one batch	In one batch
GSM2459176	AS_D4 rep 3	8784170061_A	7.8	2.06	258.9	2.14	2.27	03/06/2013_Batch1	In one batch	In one batch	In one batch
GSM2459177	AS_D6 rep 1	8762536052_E	7.1	1.97	658.79	2.25	2.38	03/06/2013_Batch2	In one batch	In one batch	In one batch
GSM2459178	AS_D6 rep 2	8762536135_A	6.5	2.24	737.49	2.23	2.38	03/06/2013_Batch2	In one batch	In one batch	In one batch
GSM2459179	AS_D6 rep 3	8784170059_B	7.2	2.54	669.17	2.28	2.41	03/06/2013_Batch2	In one batch	In one batch	In one batch
GSM2459180	AS_D8 rep 1	8762536084_D	7.2	1.15	1112.8	2.18	2.26	03/06/2013_Batch2	In one batch	In one batch	In one batch
GSM2459181	AS_D8 rep 2	8762536135_F	7	1.94	415.4	2.19	2.4	03/06/2013_Batch2	In one batch	In one batch	In one batch
GSM2459182	AS_D8 rep 3	8784170059_E	7.4	3.35	625.92	2.29	2.39	03/06/2013_Batch2	In one batch	In one batch	In one batch
GSM2459183	AS_D10 rep 1	8762536052_C	7.9	0.18	2845.83	2.07	2.16	03/06/2013_Batch2	In one batch	In one batch	In one batch
GSM2459184	AS_D10 rep 2	8762536135_D	7.8	0.21	3054.76	2.07	2.16	03/06/2013_Batch2	In one batch	In one batch	In one batch
GSM2459185	AS_D10 rep 3	8784170061_B	7.7	0.22	3857.18	1.88	1.95	03/06/2013_Batch2	In one batch	In one batch	In one batch
GSM2459186	AS_D12 rep 1	8762536072_C	7.4	0.94	4074.58	1.75	1.82	03/06/2013_Batch2	In one batch	In one batch	In one batch
GSM2459187	AS_D12 rep 2	8762536084_E	7.2	0.97	3442.65	2.02	2.1	03/06/2013_Batch2	In one batch	In one batch	In one batch
GSM2459188	AS_D12 rep 3	8784170061_C	7.9	1.02	3104.6	2.05	2.14	03/06/2013_Batch2	In one batch	In one batch	In one batch

**Online-only Table 2:** Batch information, RNA quality and concentration and related GEO accession numbers for PcCB blood (GSE93631^19^).

Series GSE93631	PRJNA361313		Bioanalyser		Nanodrop							
GEO accession ID	Sample Name in GEO	BeadChip No.	RIN	rRNA 28 s/18 s	[ng/ul]	260/280	260/230	RNA isolation batch	Comments	Globin mRNA reduction	cRNA preparation	BeadChip hybridasation
GSM2459189	CB_naive_D0 rep 1	8762536055_D	9.5	1.9	317.8	2.11	2.2	16/10/2013_Batch1		In one batch	In one batch	In one batch
GSM2459190	CB_naive_D0 rep 2	8762536056_E	9.3	1.7	248.7	2.05	2.2	16/10/2013_Batch1		In one batch	In one batch	In one batch
GSM2459191	CB_naive_D0 rep 3	8762536079_E	9.4	1.6	251.47	2.04	2.22	16/10/2013_Batch1		In one batch	In one batch	In one batch
GSM2459192	CB_naive_D12 rep 1	8762536054_F	9.9	1.9	331.23	2.1	2.2	16/10/2013_Batch2		In one batch	In one batch	In one batch
GSM2459193	CB_naive_D12 rep 2	8762536055_E	9.1	1.6	331.11	2.11	2.27	16/10/2013_Batch2		In one batch	In one batch	In one batch
GSM2459194	CB_naive_D12 rep 3	8762536056_A	9.6	1.9	537.73	2.11	2.21	16/10/2013_Batch2		In one batch	In one batch	In one batch
GSM2459195	CB_D2 rep 1	8762536049_B	9.4	1.8	324.98	2.11	2.29	16/10/2013_Batch2		In one batch	In one batch	In one batch
GSM2459196	CB_D2 rep 2	8762536054_C	9.7	2	334.51	2.13	2.24	16/10/2013_Batch2		In one batch	In one batch	In one batch
GSM2459197	CB_D2 rep 3	8762536055_F	9.4	1.7	285.15	2.13	2.28	16/10/2013_Batch2		In one batch	In one batch	In one batch
GSM2459198	CB_D2 rep 4	8762536079_C	9.2	1.7	210.81	2.12	2.22	16/10/2013_Batch2		In one batch	In one batch	In one batch
GSM2459199	CB_D4 rep 1	8762536049_F	9.6	2.4	437.07	2.11	2.25	16/10/2013_Batch1		In one batch	In one batch	In one batch
GSM2459200	CB_D4 rep 2	8762536054_D	9.5	2.5	237.53	2.1	2.23	16/10/2013_Batch1		In one batch	In one batch	In one batch
GSM2459201	CB_D4 rep 3	8762536056_B	10	2.1	295.06	2.08	2.15	16/10/2013_Batch1		In one batch	In one batch	In one batch
GSM2459202	CB_D4 rep 4	8762536078_A	10	2.1	245.02	2.08	2.25	16/10/2013_Batch1		In one batch	In one batch	In one batch
GSM2459203	CB_D6 rep 1	8762536055_A	N/A	0.9	819.77	2.26	2.55	16/10/2013_Batch1	Parasite rRNA detected	In one batch	In one batch	In one batch
GSM2459204	CB_D6 rep 2	8762536056_F	N/A	1.3	821.73	2.31	2.59	16/10/2013_Batch1	Parasite rRNA detected	In one batch	In one batch	In one batch
GSM2459205	CB_D6 rep 3	8762536079_A	N/A	1.7	427.12	2.17	2.42	16/10/2013_Batch1	Parasite rRNA detected	In one batch	In one batch	In one batch
GSM2459206	CB_D6 rep 4	8762536097_B	N/A	0.6	713.05	2.29	2.58	16/10/2013_Batch1	Parasite rRNA detected	In one batch	In one batch	In one batch
GSM2459207	CB_D8 rep 1	8762536049_D	N/A	1.6	1512.02	2.2	2.56	16/10/2013_Batch3	Parasite rRNA detected	In one batch	In one batch	In one batch
GSM2459208	CB_D8 rep 2	8762536054_E	N/A	1.2	635.79	2.34	2.6	16/10/2013_Batch3	Parasite rRNA detected	In one batch	In one batch	In one batch
GSM2459209	CB_D8 rep 3	8762536078_D	N/A	1.1	927.2	2.22	2.59	16/10/2013_Batch3	Parasite rRNA detected	In one batch	In one batch	In one batch
GSM2459210	CB_D8 rep 4	8762536097_F	N/A	1.4	1145.57	2.25	2.56	16/10/2013_Batch3	Parasite rRNA detected	In one batch	In one batch	In one batch
GSM2459211	CB_D10 rep 1	8762536049_C	10	2.1	1004.02	2.13	2.18	16/10/2013_Batch3		In one batch	In one batch	In one batch
GSM2459212	CB_D10 rep 2	8762536056_C	10	2	1969.42	2.1	2.22	16/10/2013_Batch3		In one batch	In one batch	In one batch
GSM2459213	CB_D10 rep 3	8762536078_B	10	2.1	3733.01	1.93	1.98	16/10/2013_Batch3		In one batch	In one batch	In one batch
GSM2459214	CB_D10 rep 4	8762536079_D	10	2.1	2197.77	2.09	2.18	16/10/2013_Batch3		In one batch	In one batch	In one batch
GSM2459215	CB_D12 rep 1	8762536049_E	9.9	1.8	4052.15	1.77	1.84	16/10/2013_Batch3		In one batch	In one batch	In one batch
GSM2459216	CB_D12 rep 2	8762536078_C	9.8	1.4	4284.33	1.52	1.56	16/10/2013_Batch3		In one batch	In one batch	In one batch
GSM2459217	CB_D12 rep 3	8762536079_B	9.9	1.9	2436.88	2.09	2.21	16/10/2013_Batch3		In one batch	In one batch	In one batch
GSM2459218	CB_D9 rep 1	8762536055_B	10	2.5	825.61	2.11	2.26	16/10/2013_Batch2		In one batch	In one batch	In one batch
GSM2459219	CB_D9 rep 2	8762536097_D	10	2.1	1264.37	2.1	2.22	16/10/2013_Batch2		In one batch	In one batch	In one batch
GSM2459220	CB_D9 rep 3	8762536078_F	10	2.3	675.76	2.12	2.25	16/10/2013_Batch2		In one batch	In one batch	In one batch
GSM2459221	CB_D9 rep 4	8762536054_A	10	2.4	416.6	2.1	2.24	16/10/2013_Batch2		In one batch	In one batch	In one batch

**Online-only Table 3:** Batch information, RNA quality and concentration and related GEO accession numbers for PcAS spleen (GSE123391^20^).

Series GSE123391	PRJNA508619		Caliper LabChip		Nanodrop					
GEO accession ID	Sample Name in GEO	BeadChip No.	RQS	rRNA 28 s/18 s	[ng/ul]	260/280	260/230	RNA isolation batch	cRNA preparation	BeadChip hybridasation
GSM3502544	AS-naive-day0-rep1	9440690022_B	7.6	2.66	678.78	2.07	2.31	31/05/2013_batch1	In one batch	In one batch
GSM3502545	AS-naive-day0-rep2	9440690030_C	8.2	2.79	530.39	2.08	2.32	31/05/2013_batch1	In one batch	In one batch
GSM3502546	AS-naive-day0-rep3	9440690035_B	8.6	2.13	605.41	2.09	2.32	31/05/2013_batch1	In one batch	In one batch
GSM3502568	AS-naive-day12-rep1	9440690030_F	8.7	2.35	647.28	2.11	2.31	31/05/2013_batch1	In one batch	In one batch
GSM3502569	AS-naive-day12-rep2	9440690037_C	8.4	2.34	703.11	2.13	2.31	31/05/2013_batch1	In one batch	In one batch
GSM3502570	AS-naive-day12-rep3	9440690042_A	8.4	2.4	677.79	2.13	2.33	31/05/2013_batch1	In one batch	In one batch
GSM3502547	AS-infected-day2-rep1	9440690022_C	8.4	3.93	593.21	2.15	2.32	31/05/2013_batch1	In one batch	In one batch
GSM3502548	AS-infected-day2-rep2	9440690035_A	8.1	3.4	523.12	2.15	2.31	31/05/2013_batch1	In one batch	In one batch
GSM3502549	AS-infected-day2-rep3	9440690037_D	7.1	4.38	794.01	2.11	2.31	31/05/2013_batch1	In one batch	In one batch
GSM3502550	AS-infected-day2-rep4	9440690042_C	8.4	2.33	579.89	2.11	2.34	31/05/2013_batch1	In one batch	In one batch
GSM3502551	AS-infected-day4-rep1	9440690022_D	7.2	3.89	854.39	2.11	2.31	31/05/2013_batch2	In one batch	In one batch
GSM3502552	AS-infected-day4-rep2	9440690030_A	8.5	3.13	711.79	2.09	2.31	31/05/2013_batch2	In one batch	In one batch
GSM3502553	AS-infected-day4-rep3	9440690037_E	8.2	3.36	1042.2	2.1	2.31	31/05/2013_batch2	In one batch	In one batch
GSM3502554	AS-infected-day4-rep4	9440690042_B	8.8	2.66	970.62	2.1	2.31	31/05/2013_batch2	In one batch	In one batch
GSM3502555	AS-infected-day6-rep1	9440690022_E	6.9	4.47	1283.88	2.11	2.29	31/05/2013_batch2	In one batch	In one batch
GSM3502556	AS-infected-day6-rep2	9440690035_C	7.4	2.46	1766.58	2.11	2.28	31/05/2013_batch2	In one batch	In one batch
GSM3502557	AS-infected-day6-rep3	9440690042_D	7.9	2.39	2096.84	2.1	2.27	31/05/2013_batch2	In one batch	In one batch
GSM3502558	AS-infected-day6-rep4	9440690037_A	8.2	2.57	2006.02	2.1	2.25	31/05/2013_batch2	In one batch	In one batch
GSM3502559	AS-infected-day8-rep1	9440690022_F	7.7	0.98	2670.24	2.07	2.2	31/05/2013_batch2	In one batch	In one batch
GSM3502560	AS-infected-day8-rep3	9440690030_B	7.8	0.94	2911.04	2.05	2.18	31/05/2013_batch2	In one batch	In one batch
GSM3502561	AS-infected-day8-rep4	9440690035_D	7.8	1.32	2911.33	2.04	2.17	31/05/2013_batch2	In one batch	In one batch
GSM3502562	AS-infected-day10-rep1	9440690035_E	7.8	1.29	3279.66	2.01	2.11	31/05/2013_batch2	In one batch	In one batch
GSM3502563	AS-infected-day10-rep2	9440690030_D	7.7	1.3	3418.2	1.98	2.08	31/05/2013_batch2	In one batch	In one batch
GSM3502564	AS-infected-day10-rep3	9440690037_F	7.8	1.27	3034.67	2.03	2.13	31/05/2013_batch2	In one batch	In one batch
GSM3502565	AS-infected-day12-rep2	9440690030_E	8.5	2.33	2993.75	2.04	2.17	31/05/2013_batch3	In one batch	In one batch
GSM3502566	AS-infected-day12-rep3	9440690035_F	8.4	2.21	1799.39	2.09	2.25	31/05/2013_batch3	In one batch	In one batch
GSM3502567	AS-infected-day12-rep4	9440690037_B	8.6	2.17	2257.28	2.07	2.23	31/05/2013_batch3	In one batch	In one batch

**Online-only Table 4:** Batch information, RNA quality and concentration and related GEO accession numbers for PcCB spleen (GSE145781^21^).

Series GSE145781	PRJNA608201		Caliper LabChip		Nanodrop					
GEO accession ID	Sample Name in GEO	BeadChip No.	RIN	rRNA 28 s/18 s	[ng/ul]	260/280	260/230	RNA isolation batch	cRNA preparation	BeadChip hybridasation
GSM4332890	Spleen, CB-Naïve-D0.rep1	9440690042_E	8.0	10.69	481.54	2.15	2.19	06/12/2013_Batch2	In one batch	In one batch
GSM4332891	Spleen, CB-Naïve-D0.rep2	9440690065_D	7.7	13.97	503.42	2.19	2.24	06/12/2013_Batch2	In one batch	In one batch
GSM4332892	Spleen, CB-naïve-D0.rep3	9440690046_F	9.3	10.02	390.79	2.15	2.23	06/12/2013_Batch2	In one batch	In one batch
GSM4332893	Spleen, CB-Naïve-D12.rep1	9440690056_F	7.6	1.46	516.78	2.19	2.28	06/12/2013_Batch2	In one batch	In one batch
GSM4332894	Spleen, CB-Naïve-D12.rep2	9440690059_B	7.4	2.16	501.91	2.17	2.19	06/12/2013_Batch2	In one batch	In one batch
GSM4332895	Spleen, CB-Naïve-D12.rep3	9440690061_A	8.1	3.47	890.14	2.19	2.26	06/12/2013_Batch2	In one batch	In one batch
GSM4332896	Spleen, CB-D2.rep1	9440690056_B	8.5	3.48	504.5	2.14	2.25	06/12/2013_Batch1	In one batch	In one batch
GSM4332897	Spleen, CB-D2.rep2	9440690046_A	8.2	2.29	575.9	2.16	2.24	06/12/2013_Batch1	In one batch	In one batch
GSM4332898	Spleen, CB-D2.rep3	9440690061_F	8.0	3.04	527.61	2.14	2.23	06/12/2013_Batch1	In one batch	In one batch
GSM4332899	Spleen, CB-D2.rep4	9440690059_C	8.1	2.24	633.96	2.17	2.25	06/12/2013_Batch1	In one batch	In one batch
GSM4332900	Spleen, CB-D4.rep1	9440690046_B	8.0	2.29	1481.51	2.15	2.28	06/12/2013_Batch1	In one batch	In one batch
GSM4332901	Spleen, CB-D4.rep2	9440690061_C	7.1	3.93	1171.25	2.14	1.84	06/12/2013_Batch1	In one batch	In one batch
GSM4332902	Spleen, CB-D4.rep3	9440690056_E	7.9	3.29	840.35	2.17	2.24	06/12/2013_Batch1	In one batch	In one batch
GSM4332903	Spleen, CB-D4.rep4	9440690065_F	7.7	2.14	1617.19	2.15	2.27	06/12/2013_Batch1	In one batch	In one batch
GSM4332904	Spleen, CB-D6.rep1	9440690046_C	8.9	8.89	1627.51	2.14	2.22	06/12/2013_Batch1	In one batch	In one batch
GSM4332905	Spleen, CB-D6.rep2	9440690042_F	7.4	9.01	2846.56	2.13	2.23	06/12/2013_Batch1	In one batch	In one batch
GSM4332906	Spleen, CB-D6.rep3	9440690056_A	8.0	4.39	1475.99	2.16	2.25	06/12/2013_Batch1	In one batch	In one batch
GSM4332907	Spleen, CB-D6.rep4	9440690061_B	7.7	2.73	2511.74	2.14	2.23	06/12/2013_Batch1	In one batch	In one batch
GSM4332908	Spleen, CB-D8.rep1	9440690056_C	7.6	3.14	2476.91	2.15	2.22	06/12/2013_Batch1	In one batch	In one batch
GSM4332909	Spleen, CB-D8.rep2	9440690065_A	8.4	3.42	3756.04	2.03	2.13	06/12/2013_Batch1	In one batch	In one batch
GSM4332910	Spleen, CB-D8.rep3	9440690061_E	8.2	3.38	1248.69	2.15	2.22	06/12/2013_Batch1	In one batch	In one batch
GSM4332911	Spleen, CB-D8.rep4	9440690059_D	8.2	3.22	2456.41	2.16	2.24	06/12/2013_Batch1	In one batch	In one batch
GSM4332912	Spleen, CB-D9.rep1	9440690065_C	8.1	2.72	1057.83	2.16	2.22	06/12/2013_Batch2	In one batch	In one batch
GSM4332913	Spleen, CB-D9.rep2	9440690059_E	7.9	2.26	3901.31	2	2.11	06/12/2013_Batch2	In one batch	In one batch
GSM4332914	Spleen, CB-D9.rep3	9440690061_D	8.0	2.19	2278.08	2.15	2.24	06/12/2013_Batch2	In one batch	In one batch
GSM4332915	Spleen, CB-D10.rep1	9440690065_E	8.6	2.86	1628.2	2.17	2.25	06/12/2013_Batch1	In one batch	In one batch
GSM4332916	Spleen, CB-D10.rep2	9440690056_D	8.4	2.51	1162.55	2.17	2.24	06/12/2013_Batch1	In one batch	In one batch
GSM4332917	Spleen, CB-D10.rep3	9440690046_E	8.5	2.27	2509.68	2.15	2.23	06/12/2013_Batch1	In one batch	In one batch
GSM4332918	Spleen, CB-D10.rep4	9440690059_A	8.7	2.54	1224.36	2.18	2.24	06/12/2013_Batch1	In one batch	In one batch
GSM4332919	Spleen, CB-D12.rep1	9440690059_F	8.6	2.62	1172.01	2.17	2.26	06/12/2013_Batch2	In one batch	In one batch
GSM4332920	Spleen, CB-D12.rep2	9440690046_D	8.3	2.4	909.93	2.19	2.25	06/12/2013_Batch2	In one batch	In one batch
GSM4332921	Spleen, CB-D12.rep3	9440690065_B	8.6	2.62	1047.02	2.18	2.26	06/12/2013_Batch2	In one batch	In one batch

**Online-only Table 5:** Batch information, RNA quality and concentration and related GEO accession numbers for parasite RNA (GSE145634^22^).

Series GSE145634	PRJNA607775		Bioanalyser		Nanodrop							
GEO accession ID	Sample Name in GEO	BeadChip No.	RIN	rRNA 28 s/18 s	[ng/ul]	260/280	260/230	RNA isolation batch	Comments	Globin mRNA reduction	cRNA preparation	BeadChip hybridasation
GSM4322547	naïve blood rep1	8697771087_A	9	2.85	147.7	2.1	2.25	03/06/2013_Batch1		In one batch	In one batch	In one batch
GSM4322548	Infected D8 rep1	8697771087_B	7	1.94	415.4	2.19	2.4	03/06/2013_Batch2		In one batch	In one batch	In one batch
GSM4322549	Infected D8 rep2	8697771087_C	7.2	1.15	1112.8	2.18	2.26	03/06/2013_Batch2		In one batch	In one batch	In one batch
GSM4322550	naïve blood rep2	8697771087_D	8.3	2.33	244.74	2.1	2.24	03/06/2013_Batch1		In one batch	In one batch	In one batch
GSM4322551	Infected D8 rep3	8697771087_F	9.1	2.2	1050.82	2.15	2.42	31/07/2013_Batch1		In one batch	In one batch	In one batch
GSM4322552	Infected D8 rep4	8697771096_A	N/A	2.5	667.16	2.22	2.55	31/07/2013_Batch1	Parasite rRNA detected	In one batch	In one batch	In one batch
GSM4322553	naïve blood rep3	8697771096_B	8.2	2.27	164.28	2.12	2.24	03/06/2013_Batch1		In one batch	In one batch	In one batch
GSM4322554	Parasite RNA rep1	8697771096_C	N/A	1.1	351.09	2.14	2.31	31/07/2013_Batch1	Parasite rRNA detected	In one batch	In one batch	In one batch
GSM4322555	Infected D8 rep5	8697771096_D	7.4	3.35	625.92	2.29	2.39	03/06/2013_Batch2		In one batch	In one batch	In one batch
GSM4322556	Parasite RNA rep2	8697771096_E	N/A	0	248.67	2.14	2.54	31/07/2013_Batch1	Parasite rRNA detected	In one batch	In one batch	In one batch

## References

[1] Buffet PA (2011). The pathogenesis of Plasmodium falciparum malaria in humans: insights from splenic physiology. Blood.

[2] Del Portillo HA (2012). The role of the spleen in malaria. Cellular microbiology.

[3] Cunnington AJ, Riley EM, Walther M (2013). Stuck in a rut? Reconsidering the role of parasite sequestration in severe malaria syndromes. Trends Parasitol.

[4] Van den Steen PE (2013). Pathogenesis of malaria-associated acute respiratory distress syndrome. Trends Parasitol.

[5] Deroost K (2013). Hemozoin induces lung inflammation and correlates with malaria-associated acute respiratory distress syndrome. Am J Respir Cell Mol Biol.

[6] Gazzinelli RT, Kalantari P, Fitzgerald KA, Golenbock DT (2014). Innate sensing of malaria parasites. Nat Rev Immunol.

[7] Claser C (2019). Lung endothelial cell antigen cross-presentation to CD8(+)T cells drives malaria-associated lung injury. Nat Commun.

[8] Lin JW (2017). Signatures of malaria-associated pathology revealed by high-resolution whole-blood transcriptomics in a rodent model of malaria. Sci Rep.

[9] Lee, H. J. *et al*. Transcriptomic Studies of Malaria: a Paradigm for Investigation of Systemic Host-Pathogen Interactions. *Microbiol Mol Biol Rev* **82** (2018). 10.1128/MMBR.00071-17 PMC5968457 29695497

[10] Smith ML, Styczynski MP (2018). Systems Biology-Based Investigation of Host-Plasmodium Interactions. Trends Parasitol.

[11] Rothen J (2018). Whole blood transcriptome changes following controlled human malaria infection in malaria pre-exposed volunteers correlate with parasite prepatent period. PLoS One.

[12] Bediako Y (2019). Repeated clinical malaria episodes are associated with modification of the immune system in children. BMC Med.

[13] Boldt ABW (2019). The blood transcriptome of childhood malaria. EBioMedicine.

[14] Talavera-Lopez C (2019). Comparison of whole blood and spleen transcriptional signatures over the course of an experimental malaria infection. Sci Rep.

[15] Brugat T (2014). Sequestration and histopathology in Plasmodium chabaudi malaria are influenced by the immune response in an organ-specific manner. Cellular microbiology.

[16] Brugat T (2017). Antibody-independent mechanisms regulate the establishment of chronic Plasmodium infection. Nat Microbiol.

[17] Lin JW (2018). Genomic and transcriptomic comparisons of closely related malaria parasites differing in virulence and sequestration pattern. Wellcome Open Res.

[18] Du P, Kibbe WA, Lin S (2008). M. lumi: a pipeline for processing Illumina microarray. Bioinformatics.

[19] Lin J (2017). Gene Expression Omnibus.

[20] Talavera-Lopez C, Lin J, Bediako Y, Langhorne J (2019). Gene Expression Omnibus.

[21] Lin J, Langhorne J (2020). Gene Expression Omnibus.

[22] Lin J, Langhorne J (2020). Gene Expression Omnibus.

